# Onychomadesis secondary to hand-foot-and-mouth disease: report of two cases^☆^^☆☆^

**DOI:** 10.1016/j.abd.2019.06.011

**Published:** 2020-02-20

**Authors:** Juliana Polizel Ocanha Xavier, José Cândido Caldeira Xavier Junior

**Affiliations:** a Faculdade de Medicina de Botucatu, Universidade Estadual Paulista, Botucatu, SP, Brazil; b Faculdade de Medicina, Centro Universitário Católico Salesiano Auxilium, Unisalesiano, Araçatuba, SP, Brazil

Dear Editor,

Although onychomadesis secondary to hand-foot-and mouth disease (HFM) is an an uncommon clinical manifestation; it threatens parents and child caregivers. In a case series of 145 Thai patients, it occurred in 5–37% of cases, depending on the virus.^1^ HFM is more frequently caused by coxsackie virus, subtype A6, but some enterovirus and echovirus may also be involved. It is common in children below 10 years of age,^2^ and manifests with flu-like symptoms (fever, limphadenomegaly, dizziness, vomiting and discomfort) associated to canker sores in the oral mucosae and bullae on the hands and feet. Oral-faecal transmission for 30 after the resolution of symptoms and skin lesions. Nail changes often observed in HFM are Beau lines, leukonychia, and onychomadesis.^3^ Beau lines are white transverse grooves, resulting from temporary stop nail plate formation.^3^ Onychomadesis may be a more severe form of this commitment, when nail growth is interrupted for one or two weeks, resulting in detachment of the nail plate from nail bed. The new nail grows without connection to the older one, leading to splitting and detachment of the older nail. It has been proposed that this alteration may be caused by toxic direct action of the virus in the matrix or by the inflammation secondary to maceration of digital bullae.^4^ According to a case series,^1^ onychomadesis is more usual in HFM caused by Coxsackie A6 virus than other viruses. Nevertheless, a Spanish study,^5^ which investigated an onychomadesis outbreak (311 cases) showed high frequency of HFM as a possible cause (60%), a finging confirmed by identifying coxsackie and enterovirus in faecal samples^5^ Obs. adicionar referiencia subscrito Treatment is symptomatic because it is a self-limited disease with rare sequelae. We present a 3-year-old male patient (Fig. 1) and a 7-year-old female patient (Figure 2, Figure 3)with onychomadesis secondary to HFM, with history of flu-like symptoms, followed up by oral and acral lesions. Nail changes appeared around 14 days after the symptoms began in the first case and 10 days in the second one. They had complete resolution of the nail alterations, with no specific treatment and no sequelae. Parent counselling about this manifestation is necessary to avoid untimely therapeutics and unnecessary emergency consultations.

**Figure 1:**
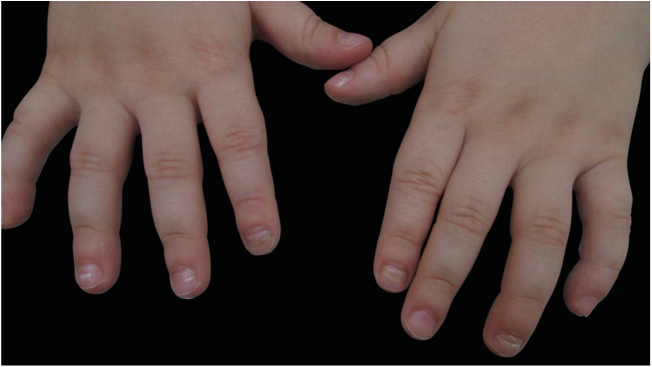
Hands from patient with 3 years old, showing Beau lines in almost every nail, and onychomadesis in second (both sides) and fourth finger.

**Figure 2:**
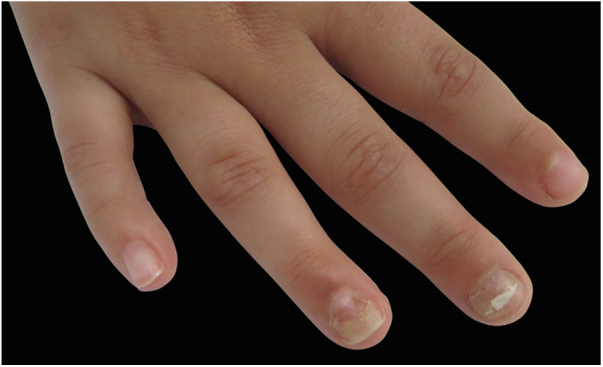
Right hand from the 7-year-old showing onychomadesis in third and fourth fingers.

**Figure 3:**
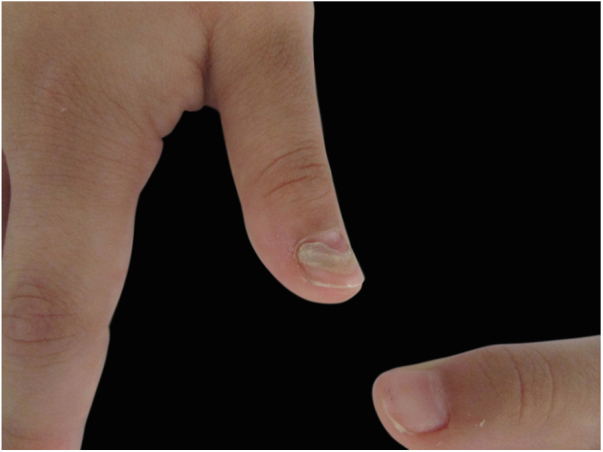
Details from first finger of the right hand from the 7-year-old patient, showing detachment of the previous nail from the newer one.

## Financial support

None declared.

## Authors’ contributions

Juliana Polizel Ocanha Xavier: Approval of the final version of the manuscript; conception and planning of the study; elaboration and writing of the manuscript; obtaining, analysis, and interpretation of the data; effective participation in research orientation; intellectual participation in the propaedeutic and/or therapeutic conduct of the studied cases; critical review of the literature; critical review of the manuscript.

José Cândido Caldeira Xavier Junior: Approval of the final version of the manuscript; critical review of the literature; critical review of the manuscript.

## Conflicts of interest

None declared.
